# Silver Wire Sutures in Wounds of the Intestines

**Published:** 1884-10-20

**Authors:** J. McF. Gaston

**Affiliations:** Atlanta, Ga.


THE
### Southern Medical Record.

EDITORS:
THOMAS S. POWELL, M.D. R. C. WORD, M.D.

*R. C. WORD, M.D., Managing Editor.*

☞All Communications and Letters on Business connected with the RECORD must be addressed to the Managing Editor.

VOL. XIV. ATLANTA, GA., OCTOBER 20, 1884. No. 10.

### ORIGINAL AND SELECTED ARTICLES.

### SILVER WIRE SUTURES IN WOUNDS OF THE INTESTINES.

BY J. McF. GASTON, M.D., ATLANTA, GA.

The elaborate paper of Dr. S. D. Gross on wounds of the intestines, and the experimental researches of Dr. C. T. Parkes on the same subject, presented to the last meeting of the American Medical Association, with the investigations of others relating to this branch of surgery, indicate a special interest in this subject at the present time. Observing that none of those who have treated of suturing the incisions or divisions of the intestinal walls have made any allusion to the advantages of the silver wire as a means of reuniting the margins in either partial or complete division of this canal, it seems proper to draw the attention of surgeons to the peculiar adaptation of this suture in wounds of the intestines. While silk and catgut are very pliant, so that in the lesser lesions of the flaccid wall of the intestinal canal they may be used in the form of continuous suture for drawing the edges together, yet this very pliancy admits of such puckering in a diagonal or longitudinal cut of the intestine as to fail in the uniform approximation of the margins throughout the entire line of the incision. If, on the other hand, the silver wire be used for making a continuous suture under the same circumstances, it can be so moulded as to serve the end of a splint in maintaining exact apposition of the edges, and yet not interfere materially with the general mobility of the intestinal canal. It will be perceived that, by the use of the blades of an ordinary dressing forceps, each stitch may be so compressed as to effect the nicest adaptation of the margins, and that the bend of the wire, being retained, must keep the parts in their relative positions after the suture is completed. Every surgeon, who has had occasion to use the silk or catgut for suturing these oblique or longitudinal incisions of the walls of the intestine, must have observed that if tension is kept up upon the tissues while the suture is being applied there is a great liability to gaping of the wound subsequently from the contraction of the longitudinal fibres of the gut, while the thread corresponds to the distended state. Again, if stitches of the continuous suture are put in while the intestine is contracted, puckering results, as the thread is too short to admit of the extension in the relaxed condition of its walls. In either case, there is more or less separation of the edges of the wound in some points of its course, and, consequently, liability to escape of gases, if not of the fluid or solid contents of the canal. If, however, the continuous splint suture of silver wire be employed, as suggested, the tissues must remain in the same relations as when it is applied, and it is only requisite to secure, so near as may be, the normal tension of the tissues on the occasion of passing the suture for the maintenance of a proper union of the borders of the wound.

Should the case be one of resection of the canal, or a transverse incision of its wall, the interrupted suture of silver wire is most appropriate; and, in this case, the accurate adjustment of the points for bringing the edges together is of moment, as all depends upon the close apposition of the margins, so that the external serous investment and the internal mucous membrane shall be in juxtaposition with their respective surfaces. While it is held by most writers that the margins shall be doubled in, so as to bring their external or peritoneal surfaces together, I am satisfied, from my observations in a case closed by me with silver wire many years ago, that the best result may be secured by precisely fitting the circular edges of the divided canal.

In the case referred to, which was reported in the medical literature of that period, a man had removed three pieces, each of some ten inches in length, from the small intestines, while under the mental hallucination of delirium tremens. When I was called to him, these three fragments were lying on the ground, and were subsequently preserved in the museum of the medical college at Charleston, S. C. The free ends of the intestine dangled out of a small aperture in the abdominal parietes, and only required to be cut off smoothly and brought together to effect the continuity of the canal. After putting in seven points of the interrupted suture of silver wire, which left the edges nicely fitted together, by twisting their ends while the point of junction was held firmly by forceps and then cutting off the wires, the union of the margins was accurately preserved. It was observed that, when the suture was completed and the intestine released, it contracted into a cord-like form at the circle of approximation, and continued in this state until the bowel was returned to the abdomen. The external wound was also closed with silver wire, and, as the subject required an opiate on account of his general condition, he was kept under its influence for more than a week. The complete recovery of this case recommends the treatment of similar accidents by the silver wire suture; and it may be further stated that, after his death from another cause some years subsequently, I was told by Dr. Chasal, of Charleston, S. C., that he made a post mortem examination of the case, remarking that the wire had disappeared through the intestinal canal, leaving but slight traces of the site of union.

It is well understood that, in sutures of the intestinal walls, plastic lymph is thrown out around the site of the stitches, so that, in cutting through the substance of the canal, the knot or loop is carried into the cavity, whether of silver, silk or other material. But should the silver wire be retained, in the continuous suture for instance, it becomes encysted and retains its place in the tissues without causing irritation. All know that it is less prone to cut its way out, and thus is preferable to silk in all cases where it is desirable that parts shall be held together firmly for a considerable time.

The catgut suture is not regarded by those having most experience with it as deserving of confidence in wounds or excisions of the intestine, and its use should be limited to closing the peritoneal abdominal incision, for which it is admirably suited.

This brief note is only designed to draw the attention of operators to the importance of studying anew the claims of the silver wire suture in their investigations of the proper mode of closing intestinal wounds.

---

Pay your subscriptions, and help us get new patrons; thereby we will be enabled to make improvements in the RECORD.

